# Celebrating Ulrik Ringborg: Multi-Omics-Based Patient Stratification for Precision Cancer Treatment

**DOI:** 10.3390/biom15050693

**Published:** 2025-05-10

**Authors:** Maria-Veronica Teleanu, Annika Schneider, Claudia R. Ball, Mathias Felix Leber, Christoph Stange, Eva Krieghoff-Henning, Katja Beck, Christoph E. Heilig, Simon Kreutzfeldt, Bernhard Kuster, Daniel B. Lipka, Stefan Fröhling

**Affiliations:** 1 Division of Translational Medical Oncology, German Cancer Research Center (DKFZ), 69120 Heidelberg, Germany; 2 National Center for Tumor Diseases (NCT), NCT Heidelberg, a Partnership Between DKFZ and Heidelberg University Hospital, 69120 Heidelberg, Germany; 3 German Cancer Consortium (DKTK), Core Center Heidelberg, 69120 Heidelberg, Germany; 4 School of Life Sciences, Technical University Munich, 85354 Freising, Germany; 5 German Cancer Consortium (DKTK), Partner Site Munich, 80336 Munich, Germany; 6 Department of Translational Medical Oncology, National Center for Tumor Diseases (NCT)/University Cancer Center Dresden, a Partnership Between DKFZ, Faculty of Medicine and University Hospital Carl Gustav Carus, Dresden University of Technology (TUD), and Helmholtz-Zentrum Dresden-Rossendorf, 01307 Dresden, Germany; 7 Translational Medical Oncology, Faculty of Medicine and University Hospital Carl Gustav Carus, Dresden University of Technology (TUD), 01069 Dresden, Germany; 8 Faculty of Biology, Dresden University of Technology (TUD), 01217 Dresden, Germany; 9 German Cancer Consortium (DKTK), Partner Site Dresden, 01307 Dresden, Germany; 10 Department of Medical Oncology, Heidelberg University Hospital, 69120 Heidelberg, Germany; 11 Clinical Cooperation Unit Virotherapy, German Cancer Research Center (DKFZ), 69120 Heidelberg, Germany; 12 Division of Personalized Medical Oncology, German Cancer Research Center (DKFZ), 69120 Heidelberg, Germany; 13 Department of Personalized Oncology, DKFZ-Hector Cancer Institute, University Medical Center Mannheim, Medical Faculty Mannheim, Heidelberg University, 68167 Mannheim, Germany; 14 Department of Personalized Oncology, University Medical Center Mannheim, Medical Faculty Mannheim, Heidelberg University, 68167 Mannheim, Germany; 15 Institute of Human Genetics, Heidelberg University, 69120 Heidelberg, Germany

**Keywords:** molecular profiling, patient stratification, precision oncology

## Abstract

Precision oncology is becoming a mainstay in the standard of care for cancer patients. Recent technological advancements have significantly lowered the cost of various tumor profiling approaches, broadening the reach of molecular diagnostics and improving patient access to precision oncology. In parallel, drug development and discovery pipelines continue to evolve, driving targeted therapeutic options forward. Yet, not all patients harboring actionable molecular alterations respond to these interventions, and existing therapies do not cover the entire spectrum of potential molecular targets. In this review, we examine the current suite of omics technologies employed in clinical settings and underscore their roles in deepening our understanding of tumor biology and optimizing patient stratification for targeted treatments. We also highlight relevant precision oncology trials and share our own experiences using multi-omics data within a molecular tumor board framework. Finally, we discuss areas for future exploration aimed at propelling precision oncology to new heights.

## 1. Introduction

Technological advances have profoundly influenced our understanding of cancer, transforming every aspect of care from diagnosis to treatment in both hematologic malignancies and solid tumors. Reflecting these developments, the World Health Organization (WHO) Classification of Tumors now incorporates specific molecular features alongside standard pathologic and immunohistochemical markers and, for some entities, is shifting toward a molecular-based taxonomy. A prime example is the fifth edition of the WHO Classification of Central Nervous System tumors, which introduced major updates underscoring the importance of molecular diagnostics, particularly DNA methylome profiling [1]. Over the past decade, advances in molecular profiling have facilitated a deeper understanding of cancer biology, propelling treatment strategies away from a “one-size-fits-all” paradigm and toward personalized, molecularly guided approaches. Consequently, seven targeted therapies have received tissue-agnostic approvals from the United States Food and Drug Administration (FDA), and four of these are also approved by the European Medicines Agency (EMA) (Table 1).

## 2. Omics Technologies Used for Guiding Precision Cancer Therapies

An illustrative example of a multi-omics program guiding precision cancer treatment is MASTER (Molecularly Aided Stratification for Tumor Eradication Research; ClinicalTrials.gov: NCT05852522). Established in 2012, MASTER unites the German Cancer Research Center (DKFZ) and all sites of the National Center for Tumor Diseases (NCT) and the German Cancer Consortium (DKTK) with their respective catchment areas in a coordinated precision oncology network. The program’s key feature is its central diagnostic pipeline, which integrates whole-genome or whole-exome sequencing (WGS/WES), RNA sequencing (RNA-seq), DNA methylation profiling, and, more recently, (phospho)proteome profiling alongside ex vivo drug sensitivity testing. By leveraging these comprehensive data layers, MASTER tailors clinical decision-making for patients with rare cancers or those diagnosed at an unusually young age with incurable common malignancies [18,19]. In the sections that follow, we examine the general strengths and limitations of these technologies and, where relevant, share our specific experiences using them in the context of MASTER.

### 2.1. Whole-Genome/Exome and RNA Sequencing

DNA-based gene panel analysis, coupled with targeted RNA-seq for fusion gene detection, has driven significant progress in precision oncology and is often viewed as the “workhorse” of molecular cancer diagnostics. This has led to the widespread integration of targeted therapies into standard treatment protocols for numerous solid tumors, with their use now increasingly shifting from later disease stages to the first-line setting. For instance, hormone receptor-positive (HR-positive) breast cancer and non-small cell lung cancer (NSCLC) are two prime examples where systematic molecular profiling has fundamentally altered therapeutic strategies [20,21]. Large clinical trials in EGFR-mutated NSCLC and PIK3CA-mutated, HR-positive, ERBB2-negative breast cancer have shown that combining targeted agents, such as kinase inhibitors, with chemotherapy or endocrine therapy is both feasible and offers notable clinical benefit over standard-of-care treatments [22,23]. Still, some patients do not respond, highlighting the importance of a more nuanced understanding of molecular tumor profiles. Moreover, most targeted therapies are approved alongside specific companion diagnostics, typically restricted to gene panels designed to identify a narrow spectrum of mutations or gene fusions, thus capturing only a fraction of potential cancer drivers or resistance mechanisms.

Studies evaluating broad profiling methods, such as WGS/WES combined with RNA-seq, have enabled us to better understand the potential of these data layers and urged us to rethink our approach to cancer treatment. Although the impact of integrating WGS/WES and RNA-seq into clinical practice on overall survival remains to be determined, these comprehensive techniques offer several advantages over gene panel sequencing and targeted RNA-based assays. In addition to identifying single-nucleotide variants (SNVs) and small insertions/deletions (indels), WGS/WES can uncover copy number alterations, tumor mutational burden (TMB), microsatellite instability (MSI), structural variants (SVs), mutational signatures, markers of homologous recombination deficiency (HRD), complex genomic rearrangements (e.g., chromothripsis, chromoanaplexy), and even viral DNA (e.g., human papilloma virus or Epstein–Barr virus) integrated into the tumor genome. Meanwhile, beyond merely guiding therapies targeting specific receptors or signaling pathways, bulk RNA-seq deconvolution can assess the intratumoral immune landscape, thereby informing more effective use of immune checkpoint inhibitors [24,25].

Likewise, bulk RNA-based functional analysis using tools, such as PROGENy (Pathway RespOnsive GENes for activitY inference), can help infer pathway activities, particularly when driver mutations are absent [26]. From the first 1310 patients analyzed in the MASTER program [19], treatment recommendations informed by RNA-based biomarkers were made for 47% of the cases, predominantly in the tyrosine kinase and immune evasion baskets, whereas somatic copy number alterations and SNVs guided treatment in 25.5% and 13.2% of the cases, respectively. By comparison, gene fusions were used in only 3.7% of the patients, and composite biomarkers in 8.7%. These findings underscore how RNA-seq can significantly expand the number of actionable targets, especially for rare cancers. Similarly, the Canadian Personalized OncoGenomics program found that among 570 patients with advanced or metastatic cancer who underwent WGS and RNA-seq, 25% received treatments solely based on RNA expression data [27]. As antibody-mediated immunotherapies continue to advance, the value of RNA-seq is expected to grow even further, enabling the detection of novel tumor antigens and accelerating the development of next-generation treatments [28].

In addition to its direct therapeutic applications, WGS or WES offers valuable insights into tumor heterogeneity and the molecular underpinnings of cancer progression and treatment resistance. A large-scale study involving 7108 genomes from primary, treatment-naive tumors and late-stage, treated tumors revealed distinct genomic features at each disease stage [29]. Specifically, metastatic tumors displayed greater genomic instability, reduced intratumor heterogeneity, and a notable increase in SVs—changes observed in more than half of the 23 cancer types examined. This rise in SVs correlated with markers of genomic instability, such as elevated ploidy and TP53 alterations. Interestingly, shifts in the overall driver landscape between primary and metastatic disease were relatively modest. However, certain cancer types showed enrichment of resistance-related driver genes at the metastatic stage (e.g., androgen receptor amplification in prostate cancer or ESR1 mutations in HR-positive breast cancer). Across multiple cancer types, three genes—*TP53, CDKN2A*, and *TERT*—were consistently enriched, underscoring their broad role in tumorigenesis.

### 2.2. DNA Methylation Profiling

Another major benefit of broad molecular profiling in clinical practice is the diagnostic utility of DNA methylome analysis, especially for rare tumors like central nervous system cancers and sarcomas, where genome-wide methylation signatures help classify tumor subtypes [29,30]. In a recent retrospective analysis of more than 3000 cases from the MASTER cohort, molecular data prompted pathologic re-evaluation for approximately 3% of the patients, with up to 90% of these findings subsequently validated by expert pathology review. The most frequent presenting diagnoses were various sarcomas (50%) and cancer of unknown primary (20%). Gene fusions triggered diagnosis in 62% of the cases, with *EWSR1::WT1* being the most relevant, leading to the identification of desmoplastic small cell round tumor in nine cases. In approximately 10% of the cases, diagnostic re-evaluation was triggered by expression or methylation patterns alone [31]. These observations highlight the diagnostic complexity of certain entities and underscore the importance of leveraging every available data layer to refine diagnoses and guide therapy. DNA methylation profiling can also be particularly useful in cases of carcinoma of unknown primary, where it aids in predicting the likely tumor type or tissue of origin [32,33,34].

Furthermore, aberrant methylation patterns serve as biomarkers for disease subtypes and potential therapeutic targets. For instance, gastrointestinal stromal tumors (GISTs) with succinate dehydrogenase (SDH) deficiency exhibit distinct hypermethylation patterns that distinguish them from classical GIST [35], and the silencing of SDHC via promoter hypermethylation has also been identified in certain GIST cases [36]. Additionally, promoter hypermethylation of MGMT, encoding O6-methylguanine-DNA methyltransferase, is used as a biomarker of MGMT silencing, which correlates with reduced responsiveness to temozolomide in glioblastoma [37]. More recently, MGMT silencing has also emerged as a potential prognostic marker in biliary tract and pancreatic cancers [38,39]. Furthermore, DNA methylation profiling can aid in identifying novel disease entities. For instance, in a recent study of spindle cell/sclerosing RMS with *FUS::TFCP2* and *EWSR1::TFCP2* fusions, distinct epigenetic patterns were observed in TFCP2-fusion RMS compared to other RMS subtypes [30].

DNA methylation profiling and classification have gained considerable traction in the study of hematopoietic malignancies. Multiple investigations have proposed new subclassifications of various hematologic neoplasms based on their methylation signatures, refining the existing frameworks and offering prognostic insights [40,41,42,43,44]. However, few of these refined classifications have transitioned into routine clinical use. A notable exception involves juvenile myelomonocytic leukemia (JMML), where three independent research groups linked aberrant DNA methylation to a more aggressive disease course [41,42,45]. Their findings led to a consensus definition of JMML “epitypes”, which serve as an independent predictor of overall survival. These epitypes can be identified from peripheral blood or bone marrow samples within approximately two to three weeks of diagnosis [46] and are currently being integrated—alongside genetic and clinical factors—into expert recommendations by the European Working Group of Myelodysplastic Syndromes (EWOG-MDS). In the United States, a prospective clinical trial has recently begun stratifying treatment intensity based on both genetic mutations and JMML epitype (NCT05849662).

The JMML example demonstrates that DNA methylation classification can be applied in a clinical setting and enrich the existing molecular datasets. However, incorporating such classification into routine practice requires more than proof-of-concept; it also demands appropriate infrastructure, technical expertise, and clinically applicable turnaround times. Recent findings presented at the Annual Meeting of the American Society of Hematology in 2024 suggest that by integrating DNA methylation data with genomic information through third-generation sequencing, comprehensive molecular diagnostics could be achieved within a matter of hours, offering a powerful approach for rapid, objective classification of myeloid neoplasms [47,48].

### 2.3. (Phospho)Proteomic Profiling

Many tumors depend on dysregulated lipid or protein kinase signaling pathways that promote proliferation, survival, and disease progression. Consequently, kinase inhibitors are among the most pivotal molecularly targeted therapies in cancer. Although certain biomarker–drug pairs (e.g., those related to *NTRK* fusions or BRAF V600E mutations) elicit deep responses, the majority of patients lack such highly actionable alterations. Further complicating matters, even patients with the same actionable biomarker and tumor type can display variable responses to targeted treatments [49,50]. Most advanced tumors present a diverse spectrum of genomic and transcriptomic features, many of which are poorly understood, making it challenging to discern which alterations truly drive tumor growth and confer resistance. This discrepancy between genotype and phenotype—likely rooted in the context-dependent oncogenicity of various mutations, alongside nongenetic mechanisms—reveals the pressing need to move beyond purely genome-focused molecular diagnostics.

Comprehensive phosphoproteome profiling of tumor tissue, which captures the functional state of the proteome at the primary site of therapeutic intervention, offers a promising way to bridge the gap between genotype and phenotype. In recent proteogenomic analyses of over 1000 untreated tumors across more than 10 cancer types, the Clinical Proteomic Tumor Analysis Consortium showed that integrating genomic data with (phospho)proteomics can delineate molecular subtypes characterized by shared oncogenic pathways, which may be particularly sensitive to targeted therapies directed at these mechanisms [51,52].

Tailoring kinase inhibitor therapies using integrative inferred kinase activity scoring [53] has shown superior efficacy in multiple preclinical studies [54,55]. In the MASTER program, we provided the first demonstration that comprehensive phosphoproteome profiling is both feasible and informative in a real-world, prospective molecular tumor board (MTB) setting. By systematically analyzing aberrant receptor tyrosine kinase activity in more than 1200 tumor specimens, we found that approximately 40% of advanced tumors exhibit nongenetic activity. This finding highlights the importance of assessing kinase activity in a patient-specific manner and underscores the value of functionally interpreting genetic abnormalities within the broader context of individual tumor biology [56,57].

Recent advances in mass spectrometry allow the quantification of approximately 8000 proteins and 30,000 phosphopeptides in single biopsy specimens. However, this deeper look into the phosphoproteome also highlights the limited biological annotation of proteins and phosphopeptides, complicating our understanding of tumor-specific signaling pathways and constraining the full potential of such data. Further research is needed to improve the functional annotation of the phosphoproteome [58] and to better characterize the target landscape of kinase inhibitors in order to enable precise molecular subtyping through phosphoproteomics and align these subtypes with suitable therapies. Ultimately, integrating longitudinal phosphoproteome profiling into clinical trials will be essential for identifying predictive biomarkers of therapeutic response.

### 2.4. Drug Sensitivity Profiling

Genetic approaches, such as WGS/WES and RNA-seq, provide a static snapshot of the tumor’s genomic landscape and may overlook how tumor dependencies shift under therapeutic pressure. To address this limitation, there is growing interest in employing additional data layers that examine therapy response in patient-derived tumor models. These functional assays can be performed directly on tumor cells obtained from surgery or biopsy or after expanding them as long-term three-dimensional organoids or spheroids. In doing so, they offer complementary information that can refine treatment decisions and enhance patient stratification.

Early studies showed that drug screening in living biobanks derived from expandable patient-derived tumor models could uncover novel vulnerabilities and therapeutic targets [59,60]. Meanwhile, functional genomic techniques enable systematic manipulation of gene expression, revealing subgroup-specific liabilities and potential therapeutic opportunities. These advances highlight the importance of integrating molecular and functional data to more precisely categorize patients into actionable therapeutic groups.

Functional testing adds a vital dimension to our understanding of tumor dependencies, revealing dynamic interactions and vulnerabilities that may be overlooked by molecular profiling alone. This insight is particularly valuable for rare cancers, where limited patient cohorts and substantial tumor heterogeneity can hamper purely molecular approaches. For instance, a recent study by Shihabi et al. [61] demonstrated that short-term sarcoma cultures grown in Matrigel enable drug sensitivity testing within just one week of tumor resection, providing timely, actionable information for clinical decision-making. Similar work in more common cancer models showed that functional profiling can uncover additional therapeutic dependencies, especially in cases where standard treatments fail or resistance emerges [62,63].

High-throughput in vitro screening strategies can further benefit patients with limited treatment options by assessing multiple therapies—or combinations—simultaneously, aiding in the prioritization of drug regimens, the identification of new therapeutic targets, and the elucidation of resistance mechanisms [64]. The EXALT1 trial (NCT03096821), conducted in advanced hematologic malignancies, illustrated the clinical feasibility and efficacy of therapy selection guided by drug response profiling, with half of the patients achieving meaningful clinical benefit, including exceptional responders [65]. Building on these results, the EXALT2 trial will prospectively compare functional testing, genomic profiling, and standard-of-care approaches in patients with relapsed or refractory hematologic malignancies and will incorporate an interdisciplinary tumor board to discuss treatment recommendations [65].

The future of functional patient stratification will hinge on refining and scaling up these methods. Miniaturized and long-term culture systems, including organoid biobanks, are being developed to maximize the utility of limited biopsy material, especially for patients with relapsed or refractory disease. These models enable extended drug testing, the exploration of resistance mechanisms, and the evaluation of immunotherapies [61,66]. At the same time, advances in artificial intelligence (AI) are enhancing the integration of molecular and functional data, leading to more accurate predictions of therapeutic efficacy [64]. Despite these promising advances, several challenges persist. Thorough standardization is crucial when analyzing drug responses in three-dimensional tumor models, as factors like culture media, matrix stiffness, and cellular composition can all influence experimental outcomes [60,66]. Moreover, rigorous clinical validation through controlled trials is needed to establish the predictive value of functional assays and define their role in routine oncology practice.

### 2.5. Clinically Applicable Multi-Omics Workflows

**Figure 1:**
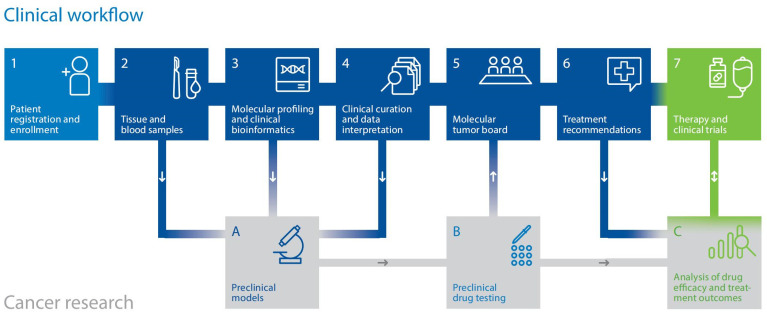
Multi-component workflow of the DKFZ/NCT/DKTK MASTER precision oncology program.

Implementing multi-omics profiling in clinical settings poses a practical challenge: integrating diverse diagnostic methods and clinical decision-making within a unified workflow that delivers standardized care on clinically feasible timelines. Through the MASTER program, launched in 2012, the combined resources of the DKFZ, NCT, and DKTK have facilitated the establishment of a specialized, quality-controlled pipeline encompassing (a) patient enrollment and consent, (b) handling of tumor and control tissue, (c) molecular analysis, (d) bioinformatic processing, biological curation, and clinical annotation of data, and (e) clinical decision-making via twice-weekly molecular tumor boards (Figure 1) [18,19,67,68].

This comprehensive framework, which has cultivated a tightly knit precision oncology network of more than 150 partners representing the spectrum of cancer care in Germany, enables the clinical application of WGS, RNA-seq, DNA methylation profiling, and, more recently, proteomic profiling and ex vivo drug testing. It has served as a pioneering model, catalyzing the broader adoption of precision oncology in Germany. Alongside using multi-omics data to guide treatment for individual patients, programs like MASTER are also laying the groundwork for molecularly stratified clinical trials. A detailed description of the workflow from sample processing through molecular analysis, data integration for treatment recommendations, and MTB discussion was recently published in the “NCT/DKFZ MASTER handbook” [18]. In brief, raw molecular data undergo bioinformatic processing using computational tools previously described by Horak et al. [19] before being evaluated by oncologists and molecular biologists for functional and clinical significance. The findings are then discussed in an interdisciplinary MTB. However, data curation, MTB preparation, and documentation still lack standardization, creating institutional variations in biomarker use and molecular data reporting. To overcome some of these limitations, our group developed the Knowledge Connector [69], a web application that integrates patients’ clinical data with various omics layers (WGS/WES, RNA-seq, methylome profiling) and precision oncology databases, enabling standardized and reproducible curation and data presentation for MTB discussions. Currently, the Knowledge Connector is used for all patients discussed in the DKFZ/NCT/DKTK MASTER MTB, and data from more than 1300 patients have been processed to date.

## 3. Precision Oncology Trials

Although broad molecular analyses have revealed an expanding array of biomarkers and potential targeted therapies, only a limited proportion of cancer patients currently benefit from these advances. There are six tissue-agnostic drug approvals in the United States and three in Europe (Table 1), and highly actionable targets occur in only a fraction of patients [2]. Basket trials have played a pivotal role by allowing patients with diverse solid tumors to receive targeted therapy based on shared molecular alterations while also identifying ineffective targets and thereby reducing unnecessary treatments. In the following, we highlight three precision oncology trials in adults and children with advanced cancers that, in our view, have significantly advanced the field.

The Drug Rediscovery Protocol (DRUP) is a prospective, non-randomized, pan-cancer clinical trial designed to assess the efficacy and safety of targeted and immunotherapies outside their approved indications, thereby broadening patient access to existing drugs [70]. Launched in the Netherlands in 2016, the trial has enrolled over 1500 patients as of November 2023, each matched to one of 36 targeted agents provided by 14 pharmaceutical companies based on molecular findings. Actionable targets are identified through routine molecular diagnostics or clinical trials, e.g., CPCT-02 (NCT01855477), which employs WGS for target identification, though a fresh biopsy is required before treatment begins. Importantly, biomarkers identified by WGS informed drug matching in 50% of the patients, with comparable rates across rare and common cancers. For the patients who underwent baseline WGS, this comprehensive approach revealed additional druggable biomarkers beyond those identified through routine diagnostics, including composite biomarkers such as MSI or high TMB [71,72]. The trial’s primary endpoint is the clinical benefit rate (CBR), defined as complete response, partial response, or stable disease at 16 weeks. Early results showed a 34% CBR in 215 treated patients, comprising 136 receiving targeted therapy and 79 receiving immunotherapies. A subsequent analysis of patients with rare cancers revealed a similar CBR (33%), with a notable overlap in druggable alterations between rare and non-rare cancers [71,72]. These findings underscore the advantages of drug repurposing, particularly for individuals with rare or treatment-refractory malignancies. Reflecting DRUP’s impact, a memorandum has been signed with precision oncology trials in the Nordic countries, and two European initiatives—Precision Cancer Medicine for All EU Citizens, funded through the EU4Health program, and Precision Cancer Medicine Repurposing System Using Pragmatic Clinical Trial, funded under Horizon Europe—were launched in 2023 to expand DRUP-like trial models across the European Union [70].

Another pivotal study was the NCI-MATCH trial (Molecular Analysis for Therapy Choice, NCT02465060), launched in 2015 as a signal-seeking precision medicine platform for adults with refractory cancers. Concluding in 2023, the trial enrolled more than 1500 of nearly 6000 screened patients, each assigned to one of 38 substudies based on a mandatory, centrally validated tumor sample. Over 20 drugs were tested, either as monotherapies or in combinations, and, unlike the DRUP trial, some had not yet received FDA approval. Of these 38 substudies, 8 closed due to lack of efficacy. Among screened patients, 37.6% harbored actionable mutations; across all substudies, the overall response rate (ORR) was 10.3%. To date, 27 substudies have been fully assessed, with seven meeting the primary endpoint. Notably, the BRAF V600E substudy achieved an ORR of 38%, contributing to the FDA approval of dabrafenib plus trametinib for all tumors bearing BRAF V600E mutations [73].

Like DRUP, NCI-MATCH enrolled a substantial proportion (38%) of patients with rare cancers, defined by the NCI as an incidence of 15 cases per 100,000. Molecular testing was performed at four NCI-MATCH Network Clinical Laboratories employing a 143-gene panel covering SNVs, indels, amplifications, and select translocations, complemented by an immunohistochemistry analysis for PTEN, MLH1, MSH2, and RB1 expression [74]. A key molecular insight was the frequent presence of co-mutations, often in subclones, which can drive therapeutic resistance, a finding consistent with other pan-cancer genomic analyses [29]. These observations underscore the value of implementing broad molecular profiling and targeted therapies earlier in the disease course, as well as exploring combination regimens to bolster antitumor responses. Both the DRUP and NCI-MATCH trials primarily relied on DNA-based molecular biomarkers to inform targeted therapy; however, transcriptome sequencing has also proven feasible and can further expand targeted treatment options [29].

The third trial we wish to highlight is the ZERO Childhood Cancer Precision Medicine Program PRISM (Precision Medicine for Children with Cancer) trial (NCT03336931) [75]. The multicenter study was conducted at eight pediatric cancer centers in Australia from 2017 to 2020. The primary objective was to determine how many patients could be offered molecularly guided treatment recommendations via a comprehensive precision medicine platform within a clinically relevant timeframe. Access to recommended therapies was facilitated through enrollment in clinical trials (16%), compassionate use (36%), institutional funding (33%), or other sources (15%). Therapy recommendations were informed by WGS, transcriptomic data, and DNA methylation analyses. Ultimately, 29% of the patients received the recommended targeted treatment, resulting in an ORR of 36% and a significantly improved two-year progression-free survival compared with standard-of-care (26% vs. 12%). Overall survival, however, did not differ significantly. Actionable fusions and structural variants yielded the highest benefit (ORR: 60%), whereas recommendations driven solely by RNA expression achieved an ORR of 15%. Still, treatment responses were observed across all scenarios, including cases supported only by preclinical data, underscoring the value of broad-based molecular profiling. Notably, a central MTB was instrumental in uniting all stakeholders, including treating clinicians, to discuss the molecular findings and prioritize the most viable targeted therapies.

Collectively, these trials underscore the feasibility and effectiveness of broad molecular diagnostics, including WGS or RNA-seq and molecularly guided therapy, in diverse, hard-to-treat cancer populations. They also provide models for incorporating precision medicine into routine care, highlighting the importance of international collaborations and robust infrastructure to ensure widespread accessibility. Ultimately, their findings signal a pivotal shift away from one-size-fits-all treatments and toward personalized, precision oncology strategies in both pediatric and adult settings.

## 4. Implementing Precision Therapies Through Molecular Tumor Boards

Between 2006 and 2020, the percentage of patients in the United States eligible for genome-targeted cancer therapies rose from 5% to 13.6%, while the response rate climbed from 3% to 7% [76]. Compelling evidence further indicates that patients who undergo molecular diagnostics experience improved progression-free and overall survival [77]. Yet, steadily declining costs for various sequencing technologies, the growing number of patients undergoing broad genomic analyses, and the rapid evolution of new targeted therapies create significant challenges for (a) ensuring accurate data curation and (b) determining the most effective therapy–target matches [78].

Molecular tumor boards serve as a cornerstone of precision oncology in both pediatric and adult settings [19,79,80,81]. They bring together experts from multiple disciplines to deliver quality-controlled interpretations of molecular data and to guide therapy selection for individual patients. In addition, MTBs provide the ideal venue for introducing new biomarkers and emerging omics approaches, such as (phospho)proteomics or ex vivo drug testing, into regular clinical practice. For example, within DKFZ/NCT/DKTK MASTER, we routinely offer WGS/WES, RNA-seq, (phospho)proteomics, and, when feasible, ex vivo drug testing. This comprehensive strategy has proven viable: more than 600 patients with (phospho)proteomics data have already been presented at the MTB, and in certain cases, this extra layer of information was decisive [56,57]. To support MTB workflows, clinical decision support systems have been developed that integrate patient-specific clinical and molecular data with existing knowledge, generating treatment recommendations for the MTB report [82]. At our institution, we use the Knowledge Connector [69], an in-house system that facilitates data curation, database integration, and multi-omics-based discussions, as described above.

From a therapeutic standpoint, most patients receive off-label drugs already approved for other indications. In Germany, reimbursement for off-label medications typically requires a formal MTB report endorsed by the relevant health insurance provider. Another route to targeted therapy is enrollment in molecularly driven trials, where MTBs serve as gateways for patient selection [82]. For example, TOP-ART, a randomized phase 2 trial comparing trabectedin/olaparib versus physician’s choice in tumors with HRD (NCT03127215), used a composite genomic metric—the TOP-ART score—to gauge underlying DNA repair defects. Assessment of this score through the MTB was mandatory for trial participation [83]. Along with patient recruitment, the MTB framework facilitated the introduction of a novel biomarker.

Finally, networks such as Cancer Core Europe [84], which runs the Basket of Baskets trial (NCT03767075) and the Molecular Tumor Board Portal [85], promote international collaboration. Overall, 36% of the Cancer Core Europe patients presented were assigned to one of the study arms available at the time of the MTB review [82].

## 5. Future Directions

In this section, we discuss emerging frontiers that, in our view, will have a major impact on shaping the future of precision oncology.

### 5.1. Artificial Intelligence and Machine Learning to Advance Precision Oncology

A key hurdle in precision oncology is managing and synthesizing the vast amount of data generated by various omics platforms and then integrating it with clinical information to guide therapeutic decisions. Thus far, AI-based tools have demonstrated significant promise, especially in image recognition [86], and large language models (LLMs) are increasingly being explored for oncology applications. For instance, in the MTB setting, LLMs could help locate relevant case or cohort studies that inform diagnosis, prognosis, or treatment efficacy for individual patients using both published sources and unstructured clinical databases [87]. They may also facilitate patient enrollment in suitable clinical trials by matching molecular and clinical profiles to the inclusion criteria of trial repositories.

However, several challenges remain, including safeguarding patient privacy, addressing biases in training data, and validating model accuracy across diverse clinical contexts [87]. Furthermore, LLMs in oncology are still prone to errors, underscoring the need for further refinement and rigorous validation before they can be broadly integrated into clinical workflows. In the near term, most real-world applications will likely follow a semi-automated model, in which LLM outputs are reviewed by human experts. Artificial intelligence agents powered by LLMs offer a promising avenue for overcoming some of these barriers, helping optimize and streamline data processes [88]. One example of success is TrialGPT, a recently introduced LLM-based matching tool for clinical trials, which reportedly achieves an 87.3% accuracy rate [89].

Developing functional decision support systems for precision oncology is among the most challenging tasks for AI. A notable collaborative initiative is the Cancer Core Europe Molecular Tumor Board Portal, which harmonizes and annotates patient data, ranging from omics to clinical details, to generate MTB reports for final review by a panel of experts [85]. To ensure a favorable benefit-to-harm ratio, AI-based systems for annotation and patient stratification must be validated in large-scale precision oncology trials, and their capacity to generalize across various data formats and sources needs thorough demonstration. Moreover, these continuously learning platforms introduce regulatory complexities, given the rapid pace of AI development and the need for real-time data updates. In most scenarios, at least for the foreseeable future, human expertise will remain essential for verifying the plausibility of model outputs and making final, patient-specific treatment decisions [90].

### 5.2. RNA-Based Development of Theranostic Approaches

Targeted therapies can be administered in various ways, with theranostics, particularly in genitourinary and neuroendocrine cancers, emerging as a rapidly expanding field [91]. At the same time, broad molecular analysis in rare malignancies can uncover new theranostic targets in tumor types where few treatment options exist and the druggable landscape remains largely unknown. A striking example from the MASTER program involved identifying elevated expression of fibroblast-activating protein alpha (FAPα) in solitary fibrous tumors (SFTs), a rare sarcoma subtype that displayed the highest FAPα expression among all entities examined. This discovery prompted the use of FAP-targeted nuclear medicine diagnostics and treatment in patients with advanced SFT, leading to disease control in 82% of cases and a median progression-free survival of 227 days. Notably, immunohistochemical analysis confirmed the high FAPα expression observed via RNA analysis [92]. These encouraging outcomes support the broader application of RNA expression-driven nuclear medicine diagnostics and therapy across multiple tumor types and warrant further integration into clinical trials and routine cancer care.

### 5.3. Exploring Virotherapy for Precision Medicine

Oncolytic measles virus (MeV)-based immunotherapies provide a prime illustration of current efforts to predict virotherapy response. In one study, Kurokawa et al. [93] used RNA-seq and gene set enrichment analysis of patient-derived glioblastoma xenografts (PDX) to identify a distinctive 22-gene “resistance” signature through diagonal linear discriminant analysis. This signature, which correlated with attenuated treatment response, was validated in additional glioblastoma and ovarian cancer PDX models, as well as in ten glioblastoma patients enrolled in a phase 1 MeV clinical trial. Constitutive interferon pathway activation emerged as a key determinant of MeV replication, independent of MeV receptor status in glioblastoma cells. However, baseline expression of the relevant virus entry receptors remains critical for successful virotherapy. Accordingly, screening for this interferon-stimulated gene resistance signature, alongside baseline receptor expression, may help identify the subset of patients most likely to benefit from oncolytic MeV-based therapy [94]. Notably, co-administration of the JAK inhibitor ruxolitinib, aimed at suppressing interferon-stimulated gene expression, successfully overcame therapy resistance in glioblastoma PDX models, underscoring the potential of RNA-based signatures to guide future virotherapy trials.

In a more recent study, Schäfer et al. [95] evaluated 14 patient-derived pancreatic ductal adenocarcinoma (PDAC) cultures for sensitivity to MeV and 4 other oncolytic agents. No single virus outperformed the others across all PDAC cultures; rather, immunological, metabolic, and proliferative pathways collectively shaped susceptibility to virotherapy. Moreover, three of the five tested viruses showed sensitivity patterns linked to the molecular subtype of PDAC. The authors proposed that combining ex vivo drug testing with transcriptomic profiling may facilitate more nuanced patient stratification for virotherapy. Adding further omics layers, e.g., WES/WGS and (phospho)proteomics, could refine predictive biomarker signatures, enabling data-driven patient selection for maximal therapeutic benefit.

### 5.4. Clinical Discovery and Reverse Translation

Precision oncology programs such as MASTER act as engines for preclinical functional and mechanistic research. By leveraging the programs’ infrastructure, biospecimens, multi-omics datasets, and clinical information, laboratory investigators can develop novel diagnostic tools, predictive biomarkers, and therapeutic targets, which are then fed back into clinical practice. A striking example is the recent work on TFCP2-rearranged RMS, a highly uncommon cancer type whose characterization was driven by observations from routine clinical care [30]. Through integrated multi-omics and functional analyses, researchers discovered that TFCP2-rearranged sarcomas have a methylation profile more similar to undifferentiated sarcomas than RMS, exhibit high genomic instability and aberrant ALK kinase expression, and harbor co-deletions of *CDKN2A/MTAP*—findings that both advance our understanding of tumor biology and lay the groundwork for exploring new therapeutic strategies.

## 6. Conclusions

In summary, precision oncology has made remarkable strides, transitioning from broad genetic profiling to increasingly integrated, multi-omics approaches that more accurately capture tumor biology. Large-scale trials and real-world programs have brought molecularly guided therapies into mainstream clinical practice, benefiting patients across a spectrum of cancer types. Yet, significant challenges remain, ranging from the sheer volume and complexity of multi-omics data to the need for validated companion diagnostics, robust AI-driven decision support, and more nuanced treatment strategies that anticipate acquired resistance. As emerging technologies, collaborative networks, and innovative trial designs continue to propel this field forward, the vision of fully personalized cancer care, where each patient receives treatment tailored to their tumor’s unique molecular profile, draws ever closer. The journey is far from over, but the progress to date signals a future ripe with possibilities for improving patient outcomes and revolutionizing oncology.

## Figures and Tables

**Table 1:** Histology-agnostic drug approvals (adapted from [2]).

Drug(s)	Biomarker/Target	Year of FDA Approval	EMA Approval	ORR	Pan-Cancer Frequency	PMID
Pembrolizumab	dMMR or	2017	Yes ^a^	40%	~3%	26028255 [3]
	MSI-high					31725351 [4]
						27157491 [5]
						29095678 [6]
						29559561 [7]
	TMB-high	2020	No	29%	20%	32919526 [8]
Dabrafenib and trametinib	BRAF V600E	2022	No	41%	3%	29072975 [9]
						35026411 [10]
						34838156 [11]
						32818466 [12]
Selpercatinib	RET fusions	2022	Yes	44%	1.5%	36108661 [13]
						32846060 [14]
Larotrectinib	NTRK fusions	2018	Yes	75%	1.6%	32105622 [15]
Entrectinib	NTRK fusions	2019	Yes	57%	1.6%	31838007 [16]
Trastuzumab Deruxtecan	ERBB2 IHC 3+	2024	No	61.3%	28%	37870536 [17]

dMMR, deficient mismatch repair; MSI, microsatellite instability; TMB, tumor mutational burden; IHC, immunohistochemistry. ^a^ colorectal carcinoma, endometrial carcinoma, gastric carcinoma, small intestine carcinoma, biliary tract cancer.

## Data Availability

No new data were created or analyzed in this study.

## Abbreviations

AI	artificial intelligence
CBR	clinical benefit rate
DKFZ	German Cancer Research Center
DKTK	German Cancer Consortium
dMMR	deficient mismatch repair
EMA	European Medicines Agency
FAP	fibroblast-activating protein
FDA	United States Food and Drug Administration
HRD	homologous recombination deficiency
IHC	immunohistochemistry
Indel	small insertions/deletions
JMML	juvenile myelomonocytic leukemia
LLM	large language model
MASTER	Molecularly Aided Stratification for Tumor Eradication Research
MeV	measles virus
MGMT	O6-methylguanine-DNA methyltransferase
MSI	microsatellite instability
MTB	molecular tumor board
NCI	National Cancer Institute
NCT	National Center for Tumor Diseases
ORR	overall response rate
PDAC	pancreas ductal adenocarcinoma
PDX	Patient-Derived Xenogaft
PROGENy	Pathway RespOnsive GENes for activity inference
RMS	Rhabdomyosarcoma
SDH	succinate dehydrogenase
SFT	solitary fibrous tumor
SNV	single-nucleotide variant
SV	structural variant
TMB	tumor mutational burden
WES	whole-exome sequencing
WGS	whole-genome sequencing
WHO	World Health Organization
